# Helical CT angiography of fenestrated stent grafting of abdominal aortic aneurysms

**DOI:** 10.2349/biij.5.2.e3

**Published:** 2009-04-01

**Authors:** Z Sun

**Affiliations:** Discipline of Medical Imaging, Department of Imaging and Applied Physics, Curtin University of Technology, Perth, Western Australia

**Keywords:** abdominal aortic aneurysm, endovascular repair, fenestrated stent graft, computed tomography, follow-up, visualization

## Abstract

Fenestrated stent grafts have been developed to treat patients with abdominal aortic aneurysms (AAA) associated with complicated aneurysm necks, such as short necks, severe angulated or poor quality necks (presence of calcification or thrombus). The technique is performed by creating an opening in the graft material so that the stent graft can be placed above the renal and other visceral branches without compromising blood perfusion to these vessels. In most situations, a supporting stent is inserted into the fenestrated vessel to provide fixation of the fenestrated vessel against stent grafts, as well as to preserve patency of the vessel. Helical CT angiography (CTA) is the preferred imaging modality in both pre-operative planning and post-procedural follow-up of fenestrated repair of AAA. The main concerns of fenestrated stent grafting lie in the following two aspects: patency of the fenestrated vessels and position of the fenestrated stents in relation to the artery branches. In this article, the author presents the clinical applications of 2D and 3D visualizations in the follow-up of patients with AAA treated with fenestrated stent grafts, with the aim of providing useful information to readers and increasing their knowledge of an increasingly used technique, fenestrated stent grafting in the treatment of AAA.

## INTRODUCTION

Endovascular aneurysm repair (EVAR) has been recognised as an effective alternative to conventional open surgery in the treatment of patients with abdominal aortic aneurysm (AAA) since it was first introduced into the clinical practice in 1991 [1, 2]. Since then, many patients have been treated with different endovascular devices, including transrenal/suprarenal fixation to enhance the stability in the proximal aneurysm neck [3-6]. However, a reasonable number of patients may remain unsuitable for such techniques on the basis of nonfavorable aortic anatomy. The main limitation to successful EVAR is due to the presence of a nonsuitable infrarenal aortic neck, which mainly includes a short (<10 mm) or angulated proximal neck (>60^°^), presence of thrombus/atheroma or severe calcification in the neck [7, 8].

The above problems limiting the endovascular repair of AAA could be solved by using a customised fenestration stent-graft design. It was initially reported in 1999, and led to successful implantation in human subjects [9-12]. Fenestrated stent grafting involves creation of an opening in the graft material. It enables the first sealing portion of the stent graft to be positioned in a more stable part of the aorta with the customized fenestrations at the exact origin of the targeted vessels. Currently, fenestrated endovascular grafts are commercially available in Australia, some European countries, and the United States.

Fenestrations are designed to be aligned with the aortic ostium of the target vessel at deployment. Thus, stent graft planning depends on careful imaging appreciation of the aortic neck anatomy. In addition, post-fenestration follow-up is of paramount importance to ensure the patency of fenestrated vessels and fenestrated stents in relation to the aortic ostium and renal/visceral perfusion. Similar to conventional endovascular repair, imaging technique plays an important role in this aspect, and helical CT angiography has been regarded as the preferred imaging modality in both pre-operative planning and post-operative follow-up. In the following sections, the author briefly introduces the technical aspect of fenestrated procedure, followed by the diagnostic applications of image visualisations arising from the CT angiography data in patients with AAA treated with fenestrated stent grafts.

## IMPLANTATION OF FENESTRATED STENT GRAFTS

**Figure 1:**
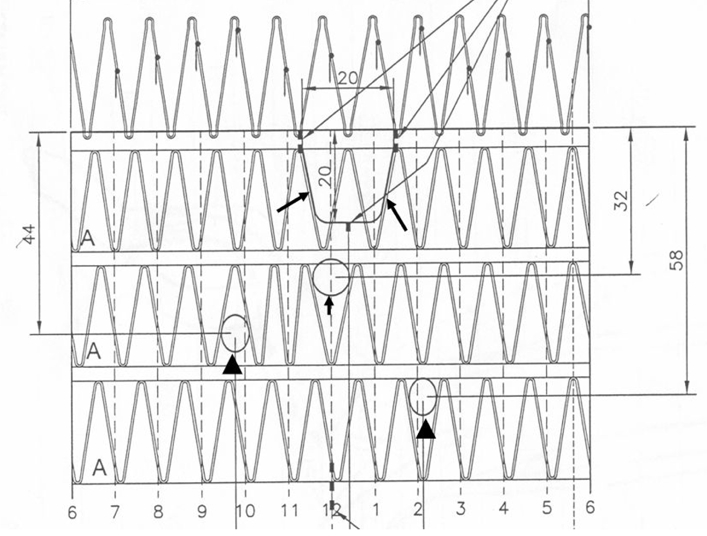
Planning diagrams for a variety of fenestration options employed in the study. A double-width fenestration (long arrow), large fenestration (short arrow) and small fenestrations (arrowheads) were planned to be implanted in the celiac axis, superior mesenteric artery and renal arteries, respectively.

The principle of stent graft fenestration is to preserve the blood flow to renal or visceral vessels and enhance stability by inserting stents into side branches to produce a durable relationship between the graft fenestration and the artery ostium. Fenestrations may be either large, or small or scallop. More recently, fenestrations have been improved by the incorporation of a nitinol circumferential ring that strengthens the edge, allowing for a more stable fixation when balloon-expandable stents are employed for accurate alignment of fenestration and artery ostia. Typically, small fenestrations, usually having a width of 6 mm and a height between 6 and 8 mm tend to be used for renal implantation and are always placed at the primary site of seal. Large and scallop fenestrations are typically used for the superior mesenteric artery and celiac axis vessels and generally are not associated with the site of seal. Large fenestrations have greater diameters between 8 and 10 mm, with a strut crossing the fenestration. Standard scallop fenestrations have a minimum width of 10 mm and a height ranging from 6 to 12 mm, while double-width scallop fenestrations measure 20 x 20 mm. Figure 1 illustrates the design options for the types of fenestrations to be employed. In most cases, the aortic artery ostia and fenestration were supported and protected by stenting [11, 12]. With stents in position, a balloon is inflated to position the stent within the renal and other visceral arteries and begin the flare.

## PATIENT DATA AND CT SCANNING PROTOCOLS

19 patients (17 male and 2 female, mean age 75 years, age range 63-86 years old) with AAA who were scheduled for fenestrated stent grafts were included in the study. Preoperative measurements required for planning of fenestrated stent grafts were performed by a group of graft planners on a separate workstation equipped with Terarecon software (www.terarecon.com). The types of fenestration used in this study include scallop, large and small fenestrations.

Multislice CT scans were performed on a High-Speed Advantage scanner (GE Medical Systems, Milwaukee, WI, USA) in 18 patients and on an Aquilion scanner (Toshiba Medical Systems, Kingsbury, UK) in the remaining one. The tube voltage and current were between 120-140 kV and 250-500 mAs, respectively. The slice thickness used in this group ranged from 0.5 to 1.25 mm in 18 patients, while in the remaining case, the slice thickness was 2.5 mm. Pitch value ranged from 0.6 to 1.0, and reconstruction interval was 0.4 mm for a section thickness of 0.5 to 0.625 mm, while for the remaining section thicknesses, it was 50% overlapping of the section thickness. All of the multislice CT angiography scans were performed with an intravenous injection of 100-120 ml non-ionic contrast media (Ultravist^R^ 300, Schering, Berlin, Germany) followed by a 40-60 ml saline chaser at a flow rate of 3-4 ml/sec. The scan was started using a bolus tracking technique with a threshold of 150 HU over baseline.

## IMAGE VISUALISATION

### 2D axial images

**Figure 2:**
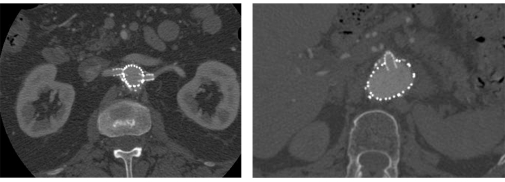
2D axial images show the small fenestrated stent inserted into the bilateral renal arteries (left) and large fenestrated stent in the superior mesenteric artery (right). Intra-aortic portion of the fenestrated stents measured 5.5 mm at the left renal stent, and 4.6 mm at the superior mesenteric artery.

2D axial images are routinely used in the post-operative follow-up of fenestrated repair of AAA. The axial images allow us to evaluate the following parameters related to the treatment outcomes: aneurysm sac diameter, fenestrated stent position in relation to the fenestrated vessels, patency of the fenestrated stents and presence of endoleaks (Fig 2). In addition, the stent protrusion into the abdominal aorta can also be accurately measured with 2D axial images, although intraluminal appearance of the stents cannot be visualised (Fig 2).

### Multiplanar reformation (MPR)

**Figure 3:**
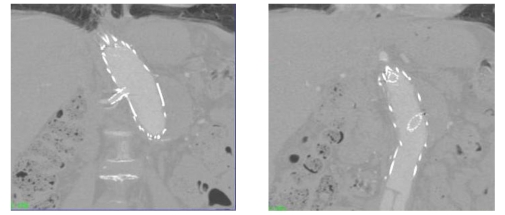
An example of coronal MPR views showing the fenestrated renal stents with an intra-aortic length of 5.2 mm (left) for the right renal stent and 17.3 mm for the left renal stent (right).

MPR is most commonly reconstructed to improve understanding of the relationship among complex anatomical structures. In comparison to 2D axial images, it provides additional information for the follow-up of fenestrated stent grafting. This is especially useful in the evaluation of tortuous vessels, and assessment of fenestrated stents in relation to the artery branches as shown in Figure 3. Moreover, intra-aortic portion of the fenestrated stents is clearly visualised and measured as demonstrated in previous studies [13, 14].

### Maximum-intensity projection (MIP)

**Figure 4:**
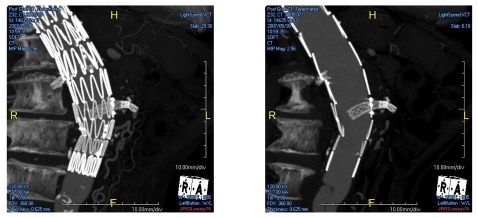
Coronal MIP shows fenestrated renal stents, however, the intra-aortic portion was difficult to visualise (left). Thin-slab MIP was generated to provide clear views of the intra-aortic fenestrated stents (right).

**Figure 5:**
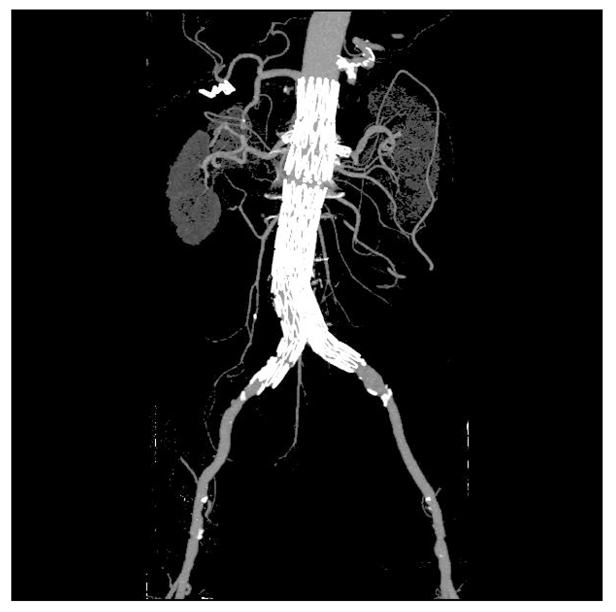
A coronal MIP demonstrating the relationship between fenestrated stent grafts and arterial branches.

MIP has been widely recognised as the most useful visualisation tool in CT angiography of EVAR as it provides angiographic-like images less invasively. High-density stent wires and contrast-enhanced vessels can be clearly displayed on MIP images. Due to overlapping of structures such as calcification and bones, thin-slab MIP is sometimes used to demonstrate the anatomical details, especially the intra-aortic portion of the fenestrated stents (Fig 4). MIP is also quite useful for follow-up of EVAR, especially the assessment of stent graft migration [15] or position of the fenestrated stent grafts in relation to the artery branches (Fig 5). The main limitation of MIP visualisation is lack of 3D relationship as it only provides 2D views of a 3D volume data.

### Volume rendering (VR)

**Figure 6:**
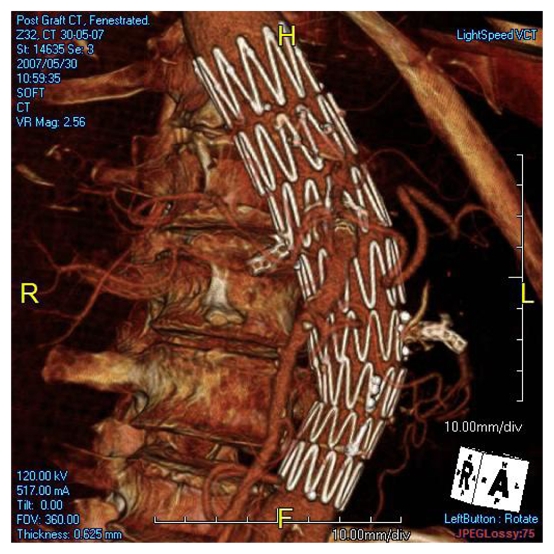
3D VR shows the relationship between fenestrated stents and vessels, with stents coded with white colour, and bones and blood vessels with red colour.

In contrast to the above-mentioned 2D visualizations, VR provides a 3D representation of the anatomical structures based on a volume dataset, since it utilises all of the information contained in the data. Therefore, 3D relationship between different structures can be easily displayed and appreciated on VR, as shown in Figure 6. Moreover, a colour can be coded to each structure including the stent graft so as to enhance understanding of the complex relationship of variable structures. Although it is believed that VR provides more meaningful images than MIP as the former clearly shows the fenestrated stents in relation to the aortic branches, especially the renal arteries (Fig 6), VR does not add more information to the original volume data.

### Virtual endoscopy (VE)

**Figure 7:**
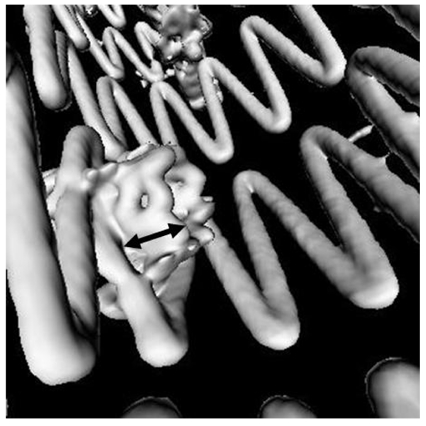
The length of the stent protruding into the aortic lumen (5.4 mm) could be accurately measured on VE visualisation.

**Figure 8:**
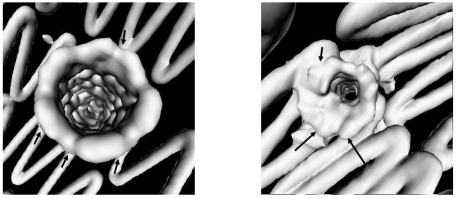
VE images provide intraluminal appearance of fenestrated renal stents which was observed as circular (left) and circular with irregularities in the lower part (right). Arrows in B indicate the flaring effect of the fenestrated procedure.

**Figure 9:**
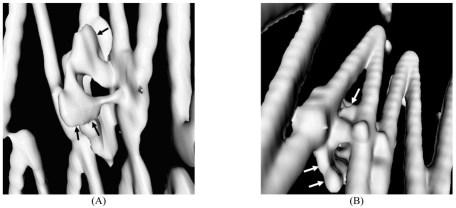
(A) is an example of a deformed fenestrated renal stent (arrows), while (B) is another example showing absence of an intraluminal portion of the fenestrated renal stent (arrows).

As part of the volume rendering, VE provides unique intravascular views of the aortic aneurysm, intraluminal stents and their relationship to the artery branches, particularly the renal arteries. Earlier studies of suprarenal fixation of stent grafts showed that VE is valuable for providing the intravascular views of the suprarenal stents in relation to the renal artery ostium which assist endovascular specialists to accurately assess the treatment outcomes of suprarenal repair of AAA [15-17]. For fenestrated stent grafting, VE is able to measure the intra-aortic portion of fenestrated stents (Fig 7) and provide the intravascular appearance of fenestrated stents (Fig 8). Early studies concluded that VE is as accurate as 2D axial and MPR views for measurements of the intra-aortic length of fenestrated stents [13, 14]. In addition, the intravascular appearance of fenestrated stents which is only acquired with VE will assist endovascular specialists to identify the post-procedural complications in terms of stent position or deformity or distortion following the fenestrated procedures. Figure 9A is an example showing the stent distortion after fenestration, while figure 9B shows that there is no intra-aortic portion of the fenestrated stents.

### 3D Stereoscopic imaging

**Figure 10:**
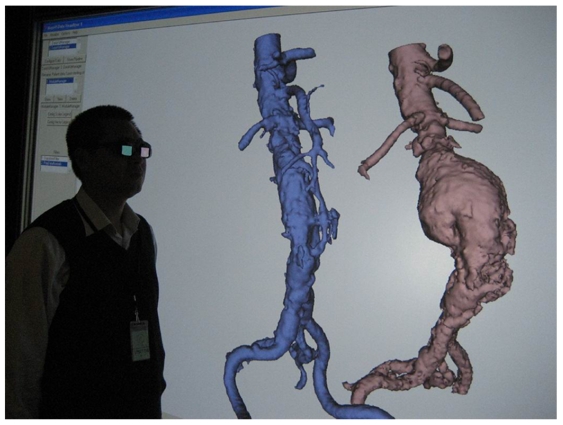
Stereo glasses are used to view CT volume data in a stereoscopic projection. Specialised hardware is required for stereoscopic viewing with the stereo glasses.

**Figure 11:**
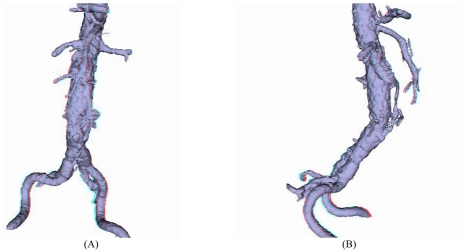
Stereoscopic views in a patient treated with fenestrated stent grafts demonstrating the presence of endoleak in relation to the aortic branches. (A) is a coronal view, while (B) is a sagittal view showing the endoleak below the right renal artery (arrows) (the reader needs red/blue glasses to appreciate the stereoscopic effect).

Despite the widely recognised value of these reconstructions, it is still difficult to appreciate the real 3D relationship between the aortic artery branches and fenestrated vessel stents due to lack of depth perception of the 2D images. To overcome the shortcoming of these displays for 3D context, stereoscopic visualisation offers promise in this aspect [18]. A stereoscopic pair of images consists of two projections of the same 3D object acquired from two slightly different viewing angles. The pair of stereoscopic images is displayed so that only the left eye sees the left projection and only the right eye sees the right projection. As a result, the observer is able to reconstruct and appreciate the 3D object mentally including the depth dimension. Readers either used complementary colour (red/blue) image pairs or stereo glasses for the acquisition of stereoscopic display (Fig 10). Early results showed that stereoscopic viewing provides additional information regarding any distortions of the fenestrated stents. Stereoscopic visualisation could be used as a complementary tool for follow-up of fenestrated stent grafting (Fig 11) [19].

## DISCUSSION AND CONCLUSION

Fenestrated stent grafting of AAA represents a further technical development compared to traditional EVAR, and it is becoming widely available worldwide. However, implantation of fenestrated stent grafts is technically demanding, and successful placement is achieved by a collaborative team of vascular surgeons and interventional radiologists. In addition, accurate pre-operative planning and post-operative follow-up is essential to ensure the success of the fenestrated procedure. Specifically, a number of 2D and 3D visualisations have been presented in this article with the aim of providing readers with information regarding the application of each visualisation tool in the follow-up of fenestrated stent grafting.

While 2D axial CT images are routinely used in clinical practice, some kind of 2D or 3D reconstructions are required to provide information which is not available with 2D axial views, but still necessary for clinical requirements. MPR is the most commonly used complementary visualisation to 2D axial images as it allows quick demonstration of the relationship between anatomical structures. As recognised widely in the literature, MIP is able to generate angiographic-like images, which are valuable for assessment of the contrast-enhanced vessels and fenestrated stents. Moreover, MIP is more accurate than axial images for assessment of stent graft migration. Although VR presents 3D information, it does not add additional information to the original axial images. The previous study showed that VR was not favoured by endovascular specialists when compared to the visualisation tools. In contrast, 3D stereoscopic view offers additional information of the fenestrated stents [19]. VE as a unique technique of providing intraluminal views of the vessel was reported to be useful for assessment of intra-aortic portion of fenestrated stents in addition to the appearance of stents.

In conclusion, as fenestrated stent grafts are used increasingly in clinical practice to treat aneurysms with complicated necks, appropriate selection of an image visualisation tool is valuable for follow-up of patients from a long-term point of view. While 2D axial images are routinely used to detect the change of aneurysm sac, patency of fenestrated stents or presence of endoleaks, post-processing reconstructions are needed for better understanding of treatment outcomes. MPR and MIP are two commonly used reconstructions complementary to axial images, and VE is reserved for patients suspected of developing fenestrated stent deformity or distortion following fenestration. With the 3D monitor being available in the market, stereoscopic view could be used as another complementary tool to traditional 2D views for follow-up of fenestrated repair.
